# Methods and Mechanisms of Photonic Disinfection

**DOI:** 10.6028/jres.126.016

**Published:** 2021-08-20

**Authors:** Daniel B. Spicer

**Affiliations:** 1 Light Sources Inc., Orange, CT 06477, USA

**Keywords:** blue light, healthcare-associated infections, ultraviolet disinfection, ultraviolet-C radiation

## Abstract

Healthcare-associated infections (HAIs) are a growing social and economic problem in the United States. A HAI is an infection that develops as a result of medical care and is typically contracted in a hospital, outpatient surgery center, nursing home, rehabilitation facility, or while receiving wound care services. One new tool to fight the increasing threat of HAIs is photonic disinfection (optical light-based disinfection). Photonic disinfection of air, surfaces, and liquids has witnessed rapid adoption within many industries (e.g., drinking water purification, wastewater treatment, indoor air purification) over the past 20 years. More recently, light-based disinfection technologies have started to make their way into hospitals, clinics, and medical centers to aid in the disinfection of air and surfaces. Two photonic disinfection methods of interest are the use of ultraviolet-C wavelengths (200 nm to 280 nm) and blue wavelengths (400 nm to 420 nm). These wavelengths of interest have been proven to be effective disinfection tools and should be put into use to augment traditional infection-prevention techniques.

## 1 Introduction

**Fig. 1:**
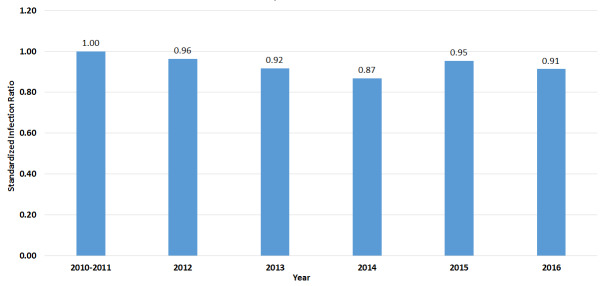
Changes over time in hospital-onset MRSA bacteremia standardized infection ratio in U.S. hospitals using 2011 as a baseline [3].

According to a 2014 report by the National Institutes of Health, approximately 2 million patients contract healthcare-associated infections (HAIs) in the United States each year, of which nearly 90,000 are estimated to die [1]. The overall direct cost of HAIs to hospitals ranges from $28 billion to $45 billion [1]. Historically, HAIs were suppressed with antibiotics and traditional chemical disinfection techniques. These traditional infection-control tools appear to be less effective on new and emerging microbial strains. This can be seen with the emergence and persistence of antibiotic-resistant bacterial strains such as Methicillin-resistant *Staphylococcus aureus* (MRSA). There has been major progress since 2005 in preventing MRSA bacteremia due to declines in hospital-onset and community-onset healthcare-associated bacteremia [2]. Unfortunately, these declines in the MRSA standardized infection ratio year over year have slowed in the United States (Fig. 1). From this information, it can be seen that MRSA and other antibiotic-resistant bacterial strains can be addressed to a limited extent with traditional treatment and control methods.

**Fig. 2:**
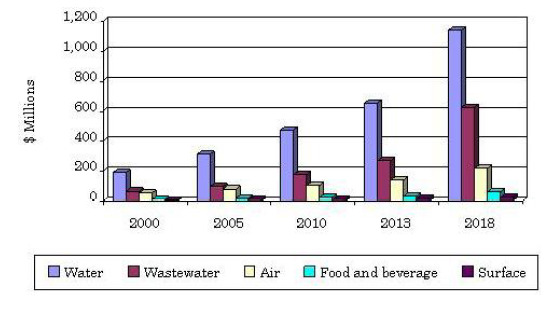
Market size and growth for UV-C disinfection equipment by end use, 2000–2018 [4].

One potential solution to drive HAI contraction rates even lower is through the deployment of photonic disinfection equipment. Photonic disinfection of air, surfaces, and liquids has witnessed rapid adoption within many industries (drinking water purification, wastewater treatment, and indoor air purification, among many others) over the past 20 years (Fig. 2). More recently, light-based disinfection technologies have started to make their way into hospitals, clinics, and medical centers. This paper explores and explains two separate photonic disinfection methods and their associated mechanisms. The first method is the use of ultraviolet-C (UV-C) wavelengths (200 nm to 280 nm) to disrupt the replication mechanism of pathogenic microorganisms. The second method is the use of blue light wavelengths (primarily 400 nm to 420 nm) to initiate a photochemical reaction resulting in cellular death to both gram-positive and gram-negative bacteria.

When discussing photonic-based disinfection methods, it is preferable to use the terms “disinfect” instead of “sterilize” and “inactivate” instead of “kill.” The typical mechanism of photonic disinfection, discussed more below, yields a metabolically living cell that is unable to replicate further, and therefore the risk of it causing infection is reduced or eliminated [5]. Thus, UV-C disinfection is a process that eliminates the regenerative capabilities of many or all pathogenic microorganisms [6]. This may not necessarily result in full sterilization, which is a process that destroys or eliminates all forms of microbial life [6]. Inactivation of a pathogen renders it biologically inert owing to loss of infectivity or antigenicity.

## 2 Mechanisms of Photonic Disinfection

Photonic disinfection is a phenomenon that is solidly grounded within the scientific field of photochemistry. When a molecule absorbs a photon of light, its electronic structure changes, and it reacts differently with other molecules. The energy absorbed from light can result in photochemical changes in the absorbing molecule, or in an adjacent molecule (e.g., photosensitization). The energy can be given off as heat, or as lower-energy light, e.g., fluorescence or phosphorescence, in order to return the molecule to its ground state [7]. The most important law of photochemistry as it pertains to photonic disinfection is the First Law of Photochemistry, also known as the Grotthuss-Draper Law, which states, “Light must be absorbed by a chemical substance in order for a photochemical reaction to take place” [8]. This implies that incident photons must directly interact with a pathogen and in turn be absorbed by the pathogen before a photochemical reaction can occur. Of equal importance in the context of photonic disinfection, the Second Law of Photochemistry states, “For each photon of light absorbed by a chemical system, only one molecule is activated for a photochemical reaction” [8]. These laws are important to keep in mind because effective photonic disinfection is dependent on light rays directly interacting with pathogens. Any area that photons do not hit will not be disinfected [9]. For example, UV-C does not penetrate through furniture or other objects that would be commonly found in a hospital room (e.g., curtains, sheets, bed frames). With these fundamentals in mind, three methods of photonic disinfection and their associated mechanisms are addressed here.

### 2.1 UV-C Light

**Fig. 3:**
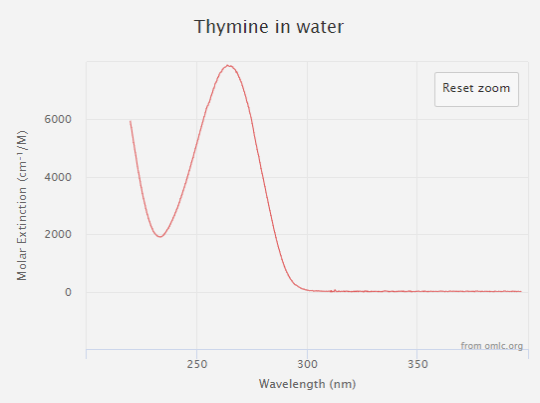
Thymine absorption spectrum [11].

One popular photonic disinfection method utilizes wavelengths in the UV-C band regime (200 nm to 280 nm [10]) to disrupt the replication mechanism of a pathogen. These wavelengths, with peak absorption occurring around 265 nm, are readily absorbed by the DNA or RNA of bacteria, virus, yeast, mold, and other pathogens of interest. Figure 3 shows the action spectra and the associated molar extinction coefficient for the DNA component thymine.

**Fig. 4:**
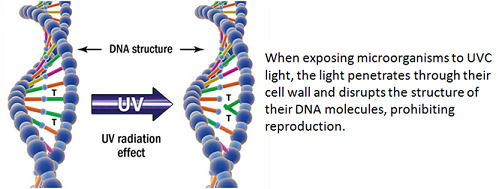
Visualization of the UV-C radiation effect on DNA and resulting thymine (T) dimer formation [12].

Extinction of thymine is important to the UV-C light method of disinfection. Once enough UV-C photonic energy has been absorbed by the DNA, a thymine dimer will be created, in which two adjacent thymine nucleotides fuse together (conceptually depicted in Fig. 4).

Once about 100 thymine dimers form within a DNA strand, the target pathogen will no longer be able to replicate and will therefore be unable to cause infection within a host [5]. Light sources that produce UV-C wavelengths include low-pressure mercury arc lamps (254 nm), UV-C light-emitting diodes (LEDs; commonly 265 nm and 280 nm), medium-pressure mercury arc lamps (polychromatic), xenon flash lamps (polychromatic), and, more recently discovered, excimer krypton chloride gas discharge lamps (222 nm). Currently, the disinfection mechanism of 222 nm excimer lamps is still under debate. Some believe that the disinfection mechanism is attributed to protein damage (which would be novel), while others attribute the disinfection mechanism to genetic damage akin to what occurs when traditional UV-C wavelengths are used. A third possibility is a combination of both genetic and protein damage. As this excimer lamp technology becomes more prolific in the marketplace and the 222 nm technologies are investigated more thoroughly, the elucidation of the mechanisms will become clearer.

**Fig. 5:**
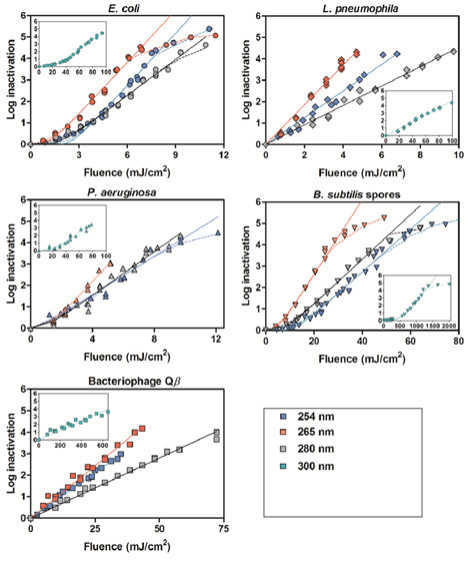
Inactivation curves for various pathogens of interest including *Escherichia coli, Legionella pneumophila, Pseudomonas aeruginosa, Bacillus subtilis and Escherichia* virus Qβ (UV-C fluence vs. log inactivation) [16]. Used with permission.

**Fig. 6:**
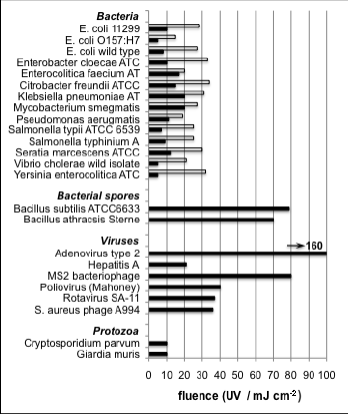
UV-C dose required for 4 log_10_ units (99.99%) inactivation of bacteria, spores, viruses, and protozoa. The bars represent conditions “in the presence of photoreactivating light” (open bars) and “in the absence of photoreactivating light” (solid bars) Note: *S. aureus* phage A994 full genus name: *Staphylococcus aureus* [17]. Used with permission.

UV-C light has proven to be an effective disinfectant of a wide variety of pathogens. Figure 5 shows the inactivation curves for five different pathogens of interest. The graphs show the fluence (UV-C energy divided by surface area required to result in an associated log inactivation of a specified pathogen of interest). Inactivation rates of 5 log_10_ units (99.999% reduction of viable pathogens) can be achieved with this photonic disinfection technique, making it a very effective tool against all HAI-causing pathogens.^1^1 5 log_10_ units refers to a 99.999% reduction, calculated as log_10_ (*N*_0_/*N*), where *N*_0_ is the initial value, and *N* is the final value.
Figure 6 shows that the fluence (also known as dose) required to achieve a common level of log inactivation varies by bacterial/viral strain. Figure 6 illustrates that this is indeed the case. Twenty-four pathogens of interest are presented in the chart in Fig. 6. Each strain requires a unique irradiance/dose to achieve a 4 log_10_ unit reduction (99.99%). Two different dosages are provided for certain bacterial strains in Fig. 6. Some microorganisms (particularly bacteria) have a repair mechanism that dissociates the thymine dimers. This process is triggered by the absorption of UV-A light and is thus called photoreactivation. Kelner [13] discovered this effect in bacteria, and Dulbecco [14, 15] discovered this effect in bacteriophages. Photoreactivation requires the pathogen in question to possess the enzyme photolase. Photoreactivation has not been observed in other types of pathogens. This repair mechanism can be overcome, but this requires a higher UV-C dosage [16].

### 2.2 Blue Light

**Fig. 7:**
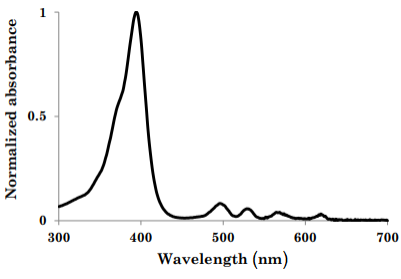
Absorbance spectra for coproporphyrin III showing the intense band of absorbance between 390 nm and 425 nm and four weaker bands between 480 nm and 700 nm [18].

**Fig. 8:**
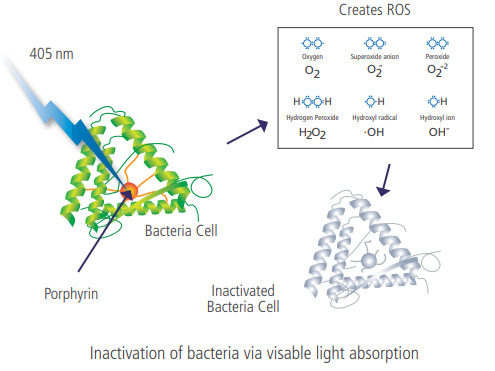
Illustration of 405 nm photonic disinfection mechanism [20].

A second, more recently discovered, photonic disinfection method utilizes blue light (400 nm to 420 nm). Blue light wavelengths (primarily 405 nm light) are absorbed by porphyrin (a molecule found in both gram-positive and gram-negative bacteria), which in turn becomes excited. Figure 7 shows the absorption properties of one common porphyrin-containing compound, coproporphyrin III. Notice the extreme absorption properties around the 405 nm wavelength. While there are a variety of common porphyrin-containing compounds found within bacteria, due to a common basic structure, all porphyrin-containing compounds share similar bands of absorption between 390 nm and 750 nm [18]. More specifically, bacterial inactivation by 405 nm light is attributed to the photoexcitation of intracellular porphyrin molecules, resulting in energy transfer and the generation of reactive oxygen species (ROS) that impart cellular oxidative damage [19]. Through a photochemical effect, the excited porphyrin molecules produce ROS. These ROS then cause intercellular oxidation damage, which in turn compromises the outer membrane of the bacterial cell, causing the cell to eventually die [19]. An illustration of this photochemical reaction can be seen in Fig. 8.

**Fig. 9:**
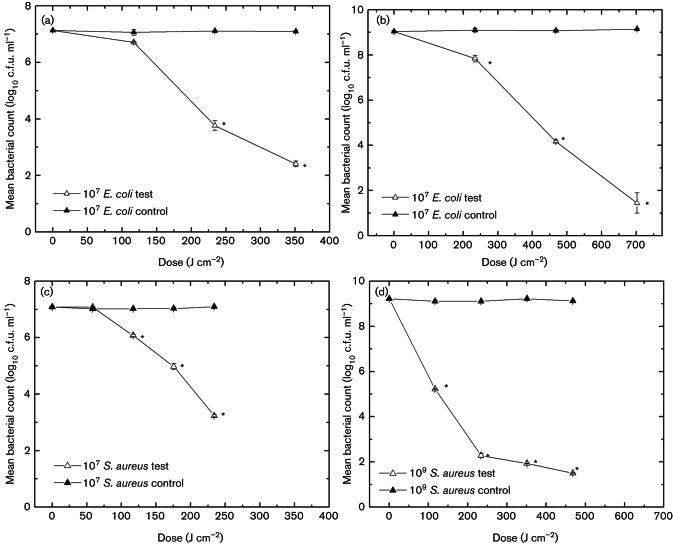
Inactivation curves for *Escherichia coli* and *Staphylococcus aureus* (405 nm dose vs. log_10_ unit inactivation) [19].

It is important to note that blue light disinfection is effective only with bacterial pathogens and is not effective on viruses because they do not contain the catalyst chemical compound porphyrin [21]. Common light sources that produce 400 nm to 420 nm blue light include phosphor-coated low-pressure mercury arc lamps and LEDs. In terms of efficacy, blue light photonic disinfection has been tested in laboratory settings, and, even though it is a relatively new technology, the results appear promising. Figure 9 shows the inactivation efficacy as it pertains to bacterial pathogens *Escherichia coli* and *Staphylococcus aureus*. From Fig. 9, dose levels of 468 J cm^−2^ (405 nm) resulted in a 7.7 log_10_ unit reduction of *Staphylococcus aureus*. For these reasons, blue light should be considered as a passive method to control the spread of bacterial-based HAIs in healthcare settings.

## 3 Methods of Photonic Disinfection

This section will focus on the implementation of the photonic disinfection technology in practical terms. Photonic disinfection methods can normally be broken up into three primary application types: surface disinfection, air disinfection, and liquid disinfection. The dominant focus of liquid disinfection is on water treatment (e.g., drinking water, wastewater, ultrapure laboratory water, treated water used in heating, ventilation, and air conditioning [HVAC] applications). In the healthcare environment, bacteria, fungi, and viruses spread mainly through person-to-person contact [22]. Because of this, liquid disinfection is not a primary focus when considering the control and reduction of HAIs. The U.S. Centers for Disease Control and Prevention have identified three HAI transmission methods: contact transmission, droplet transmission, and airborne transmission [23]. All three of these transmission methods can be interrupted by photonic disinfection of surfaces and air. Contact transmission occurs via surface-to-surface spread between a contaminated person and another person or through a contaminated object to a person [23]. Some infectious agents transmitted by the droplet route also may be transmitted by the direct and indirect contact routes. However, in contrast to contact transmission, respiratory droplets carrying infectious pathogens transmit infection when they travel directly from the respiratory tract of the infectious individual to susceptible mucosal surfaces of the recipient [23]. Airborne transmission occurs by dissemination of either airborne droplet nuclei or small particles in the respirable size range containing infectious agents that remain infective over time and distance (e.g., spores of *Aspergillus* species, and *Mycobacterium tuberculosis*). Microorganisms carried in this manner may be dispersed over long distances by air currents and may be inhaled by susceptible individuals who have not had face-to-face contact with (or have been in the same room with) the infectious individual [23]. The next two sections will provide some examples of photonic disinfection being utilized in the healthcare environment. It is important to point out that the examples provided below are not comprehensive but should give the reader some idea of how and where photonic disinfection technology can be deployed to combat HAI-causing pathogens.

### 3.1 Surface Disinfection

**Fig. 10:**
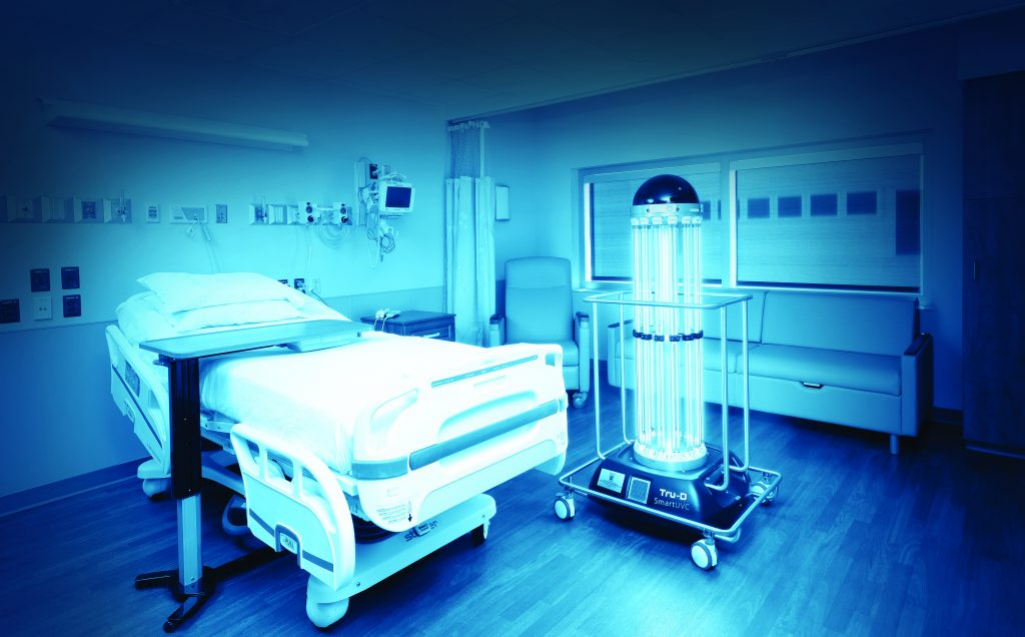
UV-C room disinfection unit [24].^2^2 Certain commercial equipment or instruments are identified in this paper for purposes of illustration. Such identification does not imply recommendation or endorsement by the National Institute of Standards and Technology, nor does it imply that the equipment or instruments identified are necessarily the best available for the purpose.

**Fig. 11:**
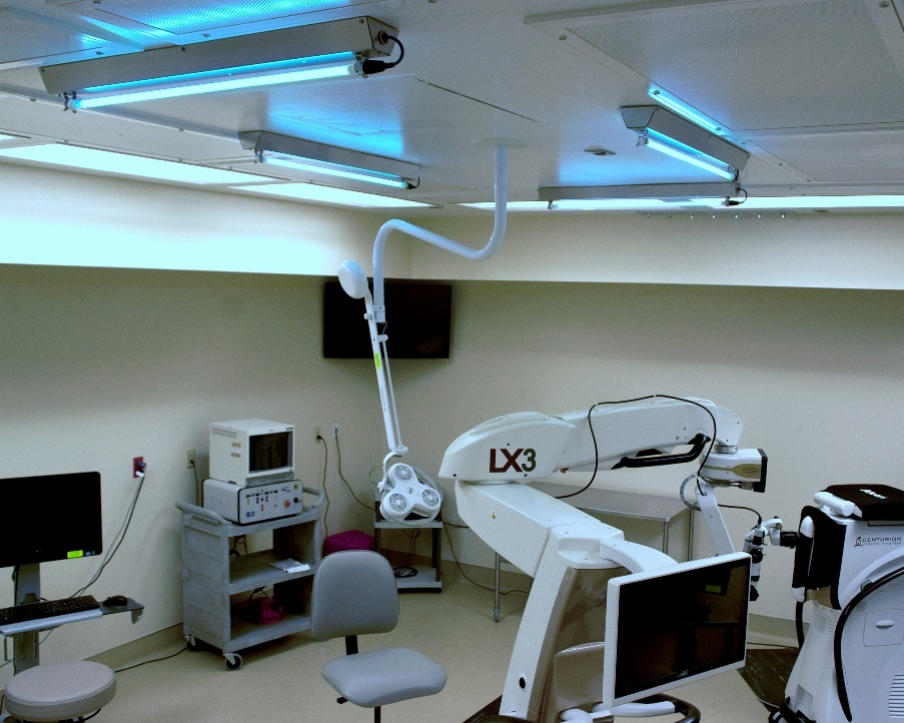
Ceiling-mounted room disinfection fixtures [25].

**Fig. 12:**
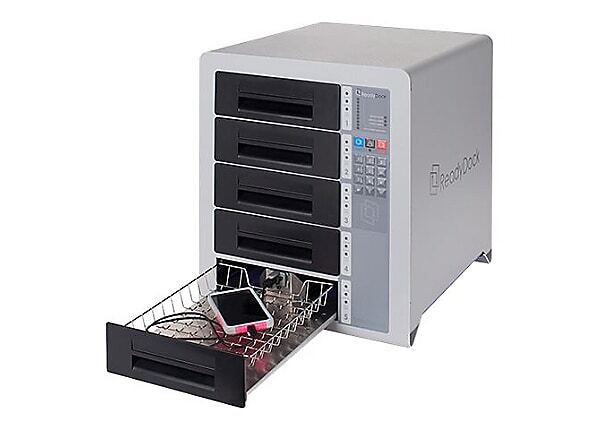
Electronic device surface disinfection unit [26].

Surface disinfection is primarily concerned with contaminated high-touch surfaces and surfaces that have had infectious aerosols land on them. Photonic disinfection of surfaces can be achieved in a variety of ways, including in-room disinfection carts (Fig. 10), ceiling-mounted disinfection fixtures (Fig. 11), and electronic device disinfection devices (Fig. 12), among others.

### 3.2 Air Disinfection

**Fig. 13:**
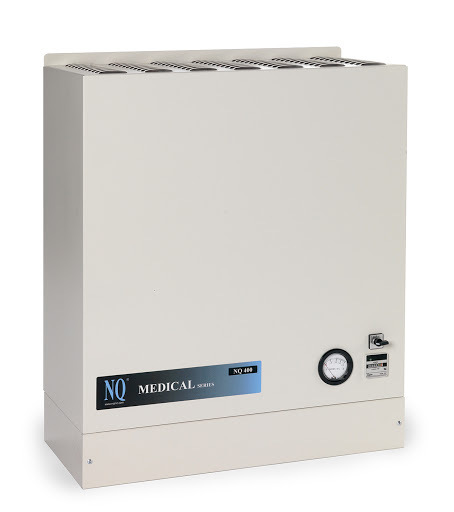
Wall-mounted UV-C air purifier [27].

**Fig. 14:**
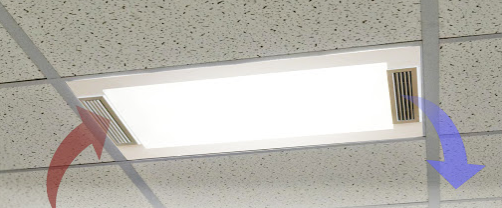
Ceiling-mounted UV-C air purifier [28].

**Fig. 15:**
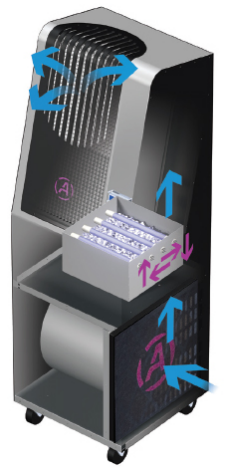
Mobile UV-C air disinfection cart [29].

Air disinfection is primarily concerned with contaminated air that contains infectious aerosolized pathogens. Two primary methods of photonic disinfection of air are in-room units and in-duct units. In-room units can be wall mounted (Fig. 13), ceiling mounted (Fig. 14), or pushed around within a mobile cart (Fig. 15).

**Fig. 16:**
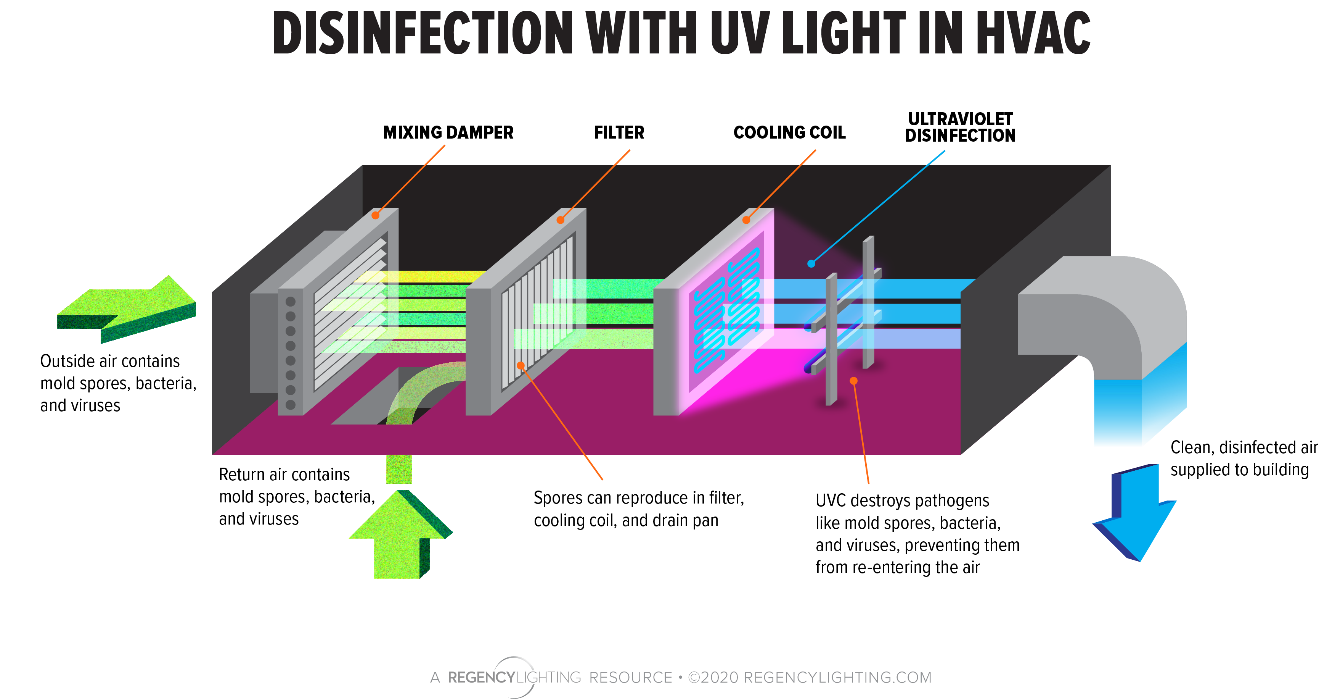
In-duct UV-C air disinfection [31]. Used with permission.

On the other hand, whole-system or in-duct units are added into HVAC ductwork. Light sources are placed at various points within the ventilation ducts or at the air conditioning coil section of an HVAC system (Fig. 16). Contaminated air is pulled/pushed through the system and passes by the disinfection light source, thereby reducing or eliminating aerosolized pathogens. UV-C disinfection is also used to treat water used in HVAC evaporative cooling systems, to prevent growth and spread of bacterial pathogens that cause Legionnaire’s Disease and similar respiratory infections [30]. It is noted that substantial power is required to disinfect rapidly moving volumes of air and that UV-C systems designed to prevent fouling of the coils often do not have adequate power to disinfect the air.

## 4 Conclusion

The social and economic costs of HAIs continue to rise with the proliferation of antibiotic-resistant pathogens such as MRSA and *Candida auris* in healthcare environments. It has become clear that traditional disinfection and hygiene techniques utilized in years past to control the spread of pathogens need to be augmented with new methods and technologies. One such technology is photonic disinfection, which has been proven to be an effective disinfectant for drinking water and wastewater during the last few decades and could now likely tackle the growing threat of HAIs in the healthcare setting. Photonic disinfection systems with UV-C wavelengths (200 nm to 280 nm) for control of viral and bacterial HAIs and blue light wavelengths (400 nm to 420 nm) for control of bacterial HAIs need to be added to the infection-prevention tool kit of the future.
